# A comprehensive welfare scoring system for graft versus host disease clinical assessment in humanised mouse models used for pharmaceutical research

**DOI:** 10.3389/fimmu.2025.1617528

**Published:** 2025-06-18

**Authors:** Alice Nowak, Rebecca Marlow, Kelli Ryan, Jean-Martin Lapointe, Daniel Sutton, Alan Sharpe, Lucy Crook, Jennifer A. Walker, Emma Little, John Peverill, Adam Holberry-Brown, Emma Wassell, Robbie McLaren-Jones, Chelsea Cavanagh, Alex Vlad Dobre, Tamara Baker, Matthew Clayton, Natasha A. Karp, Michiel Plugge, Aurélie A. Thomas, Simon J. Dovedi, Suzanne I. Sitnikova, Natalie Burrows

**Affiliations:** ^1^ Early Oncology, R&D, AstraZeneca, The Discovery Centre, Cambridge, United Kingdom; ^2^ Pathology, Clinical Pharmacology and Safety Sciences, The Discovery Centre, Cambridge, United Kingdom; ^3^ Data Sciences & Quantitative Biology, Discovery Sciences, R&D, AstraZeneca, The Discovery Centre, Cambridge, United Kingdom; ^4^ Animal Sciences and Technologies, Clinical Pharmacology and Safety Sciences, The Discovery Centre, Cambridge, United Kingdom

**Keywords:** GvHD (graft-versus-host disease), humanised animal model, immuno-oncology, 3Rs (reduce replace refine), oncology

## Abstract

Immuno-oncology drug discovery increasingly relies on humanised mouse models of cancer due to limitations of murine surrogate tools and differences between mouse and human immune systems. Graft-versus-Host Disease (GvHD) is a significant complication following xenogeneic transplantation of human immune cells into mice, limiting their lifespan and impacting the utility of these studies. Existing GvHD scoring systems inadequately capture the disease’s complexity, hampering optimal welfare management and clinical progression monitoring. We propose a comprehensive, practical scoring system for monitoring clinical signs of GvHD in humanised mice. It evaluates seven clinical signs reflecting disease complexity, sums the scores, and categorises overall GvHD severity into four stages, each with specific welfare actions. This refined tool reduces animal suffering through early detection and timely interventions, enabling mice to remain on studies where possible to maximise scientific impact. Our scoring system correlates with histological scores of GvHD-induced tissue damage across multiple organs, with liver and kidney histopathology ranking highly, unlike lung pathology. The system is reproducible among independent experimenters and versatile, effectively applied across multiple types of humanised mouse models and strains. It identifies common clinical signs including weight loss, swelling/reddening of extremities, fur condition, and posture changes, aiding users in distinguishing relevant signs. This system refines and standardises welfare decision-making, supporting the responsibility to minimise suffering when working with humanised mice.

## Introduction

Oncology drug discovery research relies extensively on humanised mouse models to gather data on the effectiveness and safety of human biological drugs (1). This reliance is attributed to the rise of immunotherapies for oncology, which require the presence of human immune system components in murine xenograft models of human cancer. Human immune cells are typically reconstituted in immunocompromised mice by engraftment of peripheral blood mononuclear cells (PBMCs), expanded T cells, antigen-specific T cells or CD34+ haematopoietic stem cells (HSCs). Graft-versus-Host Disease (GvHD) remains a significant complication following xenogeneic transplantation of human immune cells, which restricts the lifespan of mice. GvHD occurs when human donor T cells attack mouse tissues, resulting in tissue destruction and injury (2). However, the assessment of GvHD in these models is often hampered by the lack of a standardised, comprehensive scoring system which can be applied across multiple types of humanised models. This paper proposes a comprehensive and practical scoring system for GvHD in oncology humanised mouse models that supports animal welfare and therapeutic drug development. The data reported here are from mice humanised with PBMCs, expanded T cells or antigen-specific T cells, but the system has also been used to score GvHD in other models in our internal experimental work (CD34+ HSC and tumour/immune cell admix models).

In this paper, we present the first description of a GvHD scoring system specifically designed for tumour-bearing humanised mouse models, with particular focus on early clinical presentation. Several scoring systems exist for transplantation models, but these are often insufficient in identifying early or mild manifestations of GvHD, since they are mostly focused on acute GvHD, which has a different pathophysiology (3–6). Consequently, they often fall short in capturing the multifaceted nature of the disease. Holguin et al. (7) provides a method to differentiate different stages of GvHD in PBMC-humanised mice used in HIV research. However, like the other existing scoring frameworks, it focuses only on certain features of the disease, relying on broad categories that lack the granularity for comprehensive assessment, particularly in early stages of disease. This limitation poses challenges in accurately tracking progression of the disease. These challenges are particularly evident in tumour-bearing humanised models used to test human biologics, where timely mitigation strategies are needed to maximise their longevity and value in experimental settings. Therefore, there is a clear need for a more refined and detailed scoring system, such as the one presented here, that can capture the full spectrum of GvHD manifestations, specifically mild clinical signs of GvHD enabling more effective mitigation. The scoring system does so by comprehensively categorising a wide range of clinical signs into clearly defined stages that enables sensitive tracking of disease progression, particularly in mild stages of GvHD.

Our scoring system offers a precise and comprehensive tool for assessing GvHD by assigning scores to clinical signs based on severity, with higher scores indicating greater severity. After scoring all clinical signs, the total score determines overall severity, categorised into four stages from minimal to severe, each with specific welfare actions. A key advantage of scoring in distinct stages (minimal, mild, moderate and severe) is that it standardises assessment across researchers, humanised models, donors, recipient mice, sexes, and strains. This also helps minimise animal suffering through early disease detection and timely intervention to mitigate premature termination of mice before reaching scientific endpoints. For example, mice showing early weight loss can receive additional diet supplements (e.g. diet gel), helping them remain on studies until the desired endpoint is reached. This approach aligns with the principles of the 3Rs (Replacement, Reduction, and Refinement) and complies with regulatory requirements (4, 8), ensuring research involving humanised mice is conducted responsibly and ethically. The system also aims to refine humanised models and limit clinical signs by imposing time limitations on mice in mild and moderate severity categories, thereby improving welfare and minimising suffering.

A standardised scoring system for GvHD in humanised mice is essential in large-scale pharmaceutical settings as it ensures consistency and uniformity across different studies and research teams, which is crucial for the reproducibility of results. By providing a common framework, our standardised system allows for more accurate comparisons of data across experiments and facilitates collaboration between research groups.

## Materials and methods

### Mice

All procedures were ethically approved by the Animal Welfare and Ethical Review Body (AWERB). Studies were performed in accordance with the UK Animal (Scientific Procedures) Act 1986 and the EU Directive 86/609, under a UK Home Office Project License, using guidelines outlined by Workman and colleagues and the UKCCR guidance (8). Female and male NOD.Cg-Prkdcscid Il2rgtm1WjI/SzJ (NSG) mice (strain code 614) (9) were sourced from Charles River UK and NOD.Cg-Prkdcscid H2-K1b-tm1Bpe H2-Ab1g7-em1Mvw H2-D1b-tm1Bpe Il2rgtm1Wjl/SzJ (NSG MHC I/II DKO) mice (strain code 025216) (10) were sourced from The Jackson Laboratory, USA and were aged between 8 to 10 weeks. Mice were housed in Tecniplast Green Line IVC Sealsafe cages (maximum 5 mice per cage) under specific pathogen-free conditions. Cages contained irradiated aspen chip bedding, nestlet and Enviro dry nesting material, a red plastic tunnel, a cardboard house and wooden chew blocks. The facility maintained standardised environmental conditions (temperature: 20 – 26°C; relative humidity: 40 – 70%; 12/12-hour light/dark cycle). UV-treated water and RM1 rodent diet were provided ad libitum. Acclimatisation periods were two weeks for US-sourced mice and one week for UK-sourced mice. Diet supplements (such as diet gel) were given to all mice with weight loss over 8%, calculated from maximum body weight recorded on the study.

The study included 18 NSG females, 3 NSG MHC I/II DKO females, and 10 NSG MHC I/II DKO males for comparing GvHD clinical signs with histology. All tissues collected from these mice were included in this analysis. The number of mice in each GvHD severity category were minimal (n = 8), mild (n = 5), moderate (n = 4), and severe (n = 14). To enable this explorative analysis, tissues were collected opportunistically over 6 months from mice in ongoing studies. Additionally, to assess inter-operator variability, 12 female NSG-MHC I/II DKO mice were independently scored for GvHD by multiple operators. This sample size and approach were based on published recommendations for validity and inter-operator assessment, which suggest using at least 30 heterogeneous samples and involving at least 3 raters (11). While we followed these guidelines, we acknowledge the limitations of this testing and potential challenges in broader implementation. Each mouse was considered an independent experimental unit.

### Humanised mouse models of human cancer

Data was collected from 31 mice enrolled into ongoing experiments regardless of gender, strain, humanisation method, tumour model or treatment, to evaluate their GvHD scores. For humanisation, NSG or NSG MHC I/II DKO female and male mice were injected intravenously (i.v.) with either 1x10^7^ *in vitro* expanded T cells, 1x10^7^ PBMCs or 1x10^6^ antigen-specific T cells and treated with vehicle, negative control drugs or candidate drugs (**Supplementary Table 1**). See **Supplementary Methods** for humanisation, tumour implantation and welfare endpoints.

### Scoring system

**Figure 1:**
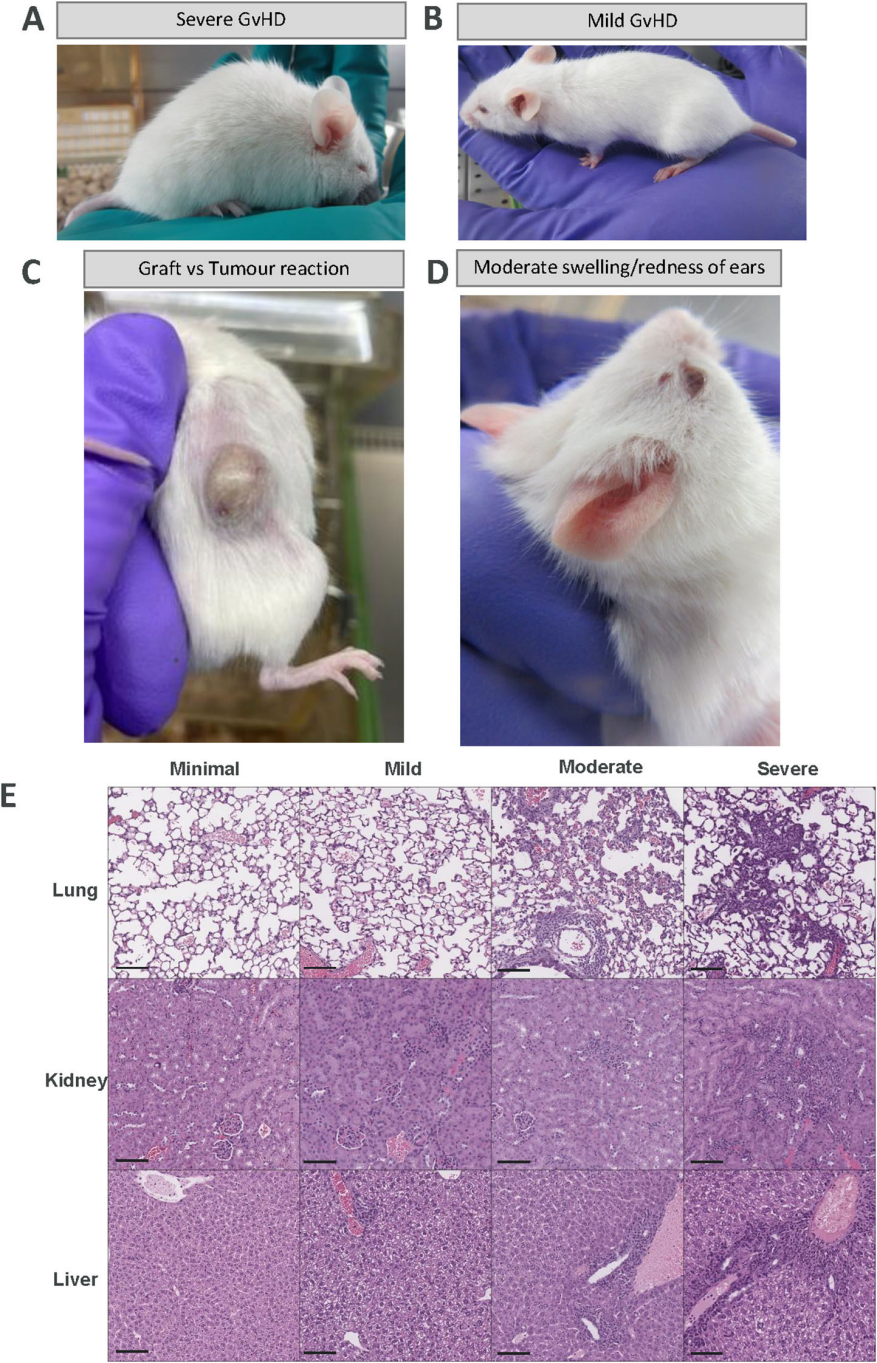
Examples of severe, mild, tumour- specific and moderate GvHD manifestations in humanised mice and representative H&E images of lung, kidney and liver. **(A)** Representative image of an overall severity score of 12 (severe). This mouse intermittently shows a hunched posture (posture score of 4), marked piloerection (fur condition score of 4), mild yellowing/paleness of extremities (inflammation score of 1) and activity in cage only when provoked (activity score of 3) meaning the mouse will be culled. **(B)** Representative image of an overall severity score of 4 (mild). This mouse shows partial piloerection (fur condition score of 1), mild swelling/redness of extremities (inflammation score of 1) and body weight loss between -10 to -14% (body weight loss score of 2) so this mouse would be culled 14 days after this score was initially observed. The animal would additionally be reassessed daily in case of worsening clinical signs. **(C)** Representative image of yellowing of the tumour indicative of a Graft-versus-tumour (GvT) reaction and **(D)** Representative image of moderate swelling/redness of ears which are common signs of early GvHD observed in humanised mice models. **(E)** Representative images of H&E sections of lung, liver and kidney from mice with GvHD from each severity category. Images show an increase in lesions and lymphocyte infiltration with increased severity from minimal to severe. Scale bar represents 100 µm.

The scoring system was developed through a collaborative effort involving Named Animal Care and Welfare Officers (NACWOs) and Named Veterinary Surgeons (NVSs), who are experts in animal welfare and severity assessment. Their expertise, along with NC3Rs guidance on welfare assessment (12) was crucial in determining the relative importance and impact of different clinical signs on animal well-being. Existing literature (3, 5, 6) and pilot studies involving daily clinical sign monitoring of PBMC-humanised female NSG mice were initially used to capture the most common signs of GvHD, relating to activity, posture, weight loss, inflammation of extremities, fur condition, diarrhoea, and abnormal breathing, examples of which can be seen in **Figures 1A–D**. Each clinical sign was assigned a score based on severity, with higher scores indicating greater severity (**Table 1**). The disparate numerical values and varying intervals between severity levels for each clinical sign reflect the differential impact on welfare and non-linear progression of severity. Some clinical signs were deemed to have a more significant effect on animal welfare than others. For instance, severe changes in posture or activity level (scored 9) were considered to have a greater impact on the animal’s overall well-being compared to diarrhoea (scored 3). The non-linear progression in scores for posture and activity (0, 1, 4, 9) reflects the expert assessment that the transition from moderate to severe in these categories represents a more dramatic decline in welfare than the transition from mild to moderate. This non-linear approach allows for more sensitive detection of critical welfare changes

**Table 1:** GvHD clinical signs scoring system used to rank severity of each sign.

Clinical sign	Observation	Score
Activity	Normal (eating/drinking/interaction with cage mates)	0
	Activity noticeably slowed in cage when unprovoked	1
	Activity in cage only when provoked	3
	No activity in cage when provoked	9
Posture	Normal posture	0
	Subdued but responsive, normal if provoked. Interacts with peers	1
	Hunched intermittently	4
	Hunched posture persistently (frozen)	9
Weight loss	No weight loss (0% from maximum weight)	0
	0-9.9% weight loss	1
	10-14.9% weight loss	2
	15-19.9% weight loss	6
	>20% weight loss	9
Inflammation of extremities (ear, muzzle, tail, paws, legs (including tumour))	No swelling	0
	Mild swelling/redness of extremities Or yellowing/paleness of extremities	1
	Moderate swelling/redness or yellowing/paleness of extremities causing some discomfort/lameness, but mouse can interact and exhibits normal behaviours within the group	4
	Severe swelling or yellowing/paleness of extremities (including tumour) causing visible discomfort and/or marked lameness	9
Fur condition	Normal fur	0
	Partial piloerection	1
	Staring coat - marked piloerection	4
	Marked piloerection with other signs of dehydration e.g. skin tenting	9
Diarrhoea	No diarrhoea	0
	Transient diarrhoea or soft stools	2
	Intermittent diarrhoea with no dehydration	3
	Continuous diarrhoea with faecal soiling of perineum or dehydration	9
Breathing abnormality	Normal	0
	Slight abnormal respiration	2
	Intermittent respiratory abnormality	5
	Continuous respiratory abnormality (laboured breathing with gasping)	9

After scoring all signs, the total score determined overall severity, which was categorised into four levels, minimal (overall scores of 0-3), mild (overall scores of 4-6), moderate (overall scores of 7-8) and severe (overall scores of ≥ 9), each with specific welfare actions, including immediate humane endpoint of a mouse found to have a severe score (**Table 2**). Time limits were implemented for the mild and moderate categories to refine animal welfare by preventing mice from remaining on study with clinical signs for prolonged periods. Mice classified under the mild category were assigned a 14-day limit, whereas those in the moderate category had a 5-day limit imposed before being culled. Mice that progressed from the mild to moderate category were monitored for up to 5 days and were culled on day 5 if this occurred before the 14-day observation period ended. Thus, the maximum monitoring period for mice progressing from mild to moderate categories was 14 days. Scores for all mice were documented daily once clinical signs appeared.

**Table 2:** GvHD clinical signs scoring system decision criteria and severity limits for each category .

Total score	Category description	Action to be taken
0 to 3	Minimal clinical signs	• Continue to monitor mice (3x/week)
4 to 6	Mild clinical signs	• Monitor closely (daily) and consult with animal welfare officer and/or laboratory animal veterinarian if required • Animals in MILD category will be monitored for up to 14 days and humanely killed at day 14 *Worsening of clinical condition at any point during the 14 days observation will be categorised as MODERATE or SEVERE*
7 to 8	Moderate clinical signs	• Monitor closely (daily) and consult with animal welfare officer and/or laboratory animal veterinarian if required • Animals in MODERATE category will be monitored for up to 5 days and humanely killed at day 5 *MOVE MILD to MODERATE* *Maximum monitoring in MILD/MODERATE is 14 days*
≥9	Severe clinical signs	Cull immediately, as risk of exceeding moderate severity

The scoring system has been adapted and refined over time, with clinical signs (such as the yellowing of tumour) and scores being added or refined based on experience across various humanised models and strains encompassing approximately 10,000 mice. These include those used for the histological scoring presented here for PBMC, expanded T cell and antigen-specific T cell models in NSG and NSG MHC I/II DKO mice, as well as other humanised models (CD34+ HSC, tumour/immune cell admix models) and strains such as BRGSF-HIS (13) and NOG (14). The reproducibility of the scoring system was evaluated by having three scientists independently score three mice of each GvHD severity category. Scoring scientists were blinded from treatment or humanisation method the mice received.

### Histology

Kidney, liver and lung were fixed in 4% formaldehyde solution and wax-embedded in paraffin. For each tissue, 4µm sections were stained with Haematoxylin and Eosin (H&E). Samples were examined histologically by a blinded board-certified veterinary pathologist with extensive experience in laboratory animal pathology, including experience with evaluation of GvHD in humanised mouse models. The tissue changes observed were determined to be GvHD-associated when they were consistent with established knowledge of GvHD pathology in humanised mice, based on the pathologist’s prior experience, as well as published literature (15, 16). For each organ, the severity of the GvHD-related lesions was scored semi-quantitatively on a scale of 0-4, based on the extent of the changes (0= no lesions, 1=minimal, 2=mild, 3= moderate, 4= marked). The scores of the three organs were added to obtain a total GvHD histological score (0-12). Example histological images of each severity category for lung, kidney and liver are provided in **Figure 1E**.

### Statistics

Data were analysed using GraphPad Prism (version 9.4.0) and R (version 4.1.0) within RStudio (version2022.02.3). The likelihood of whether increasing clinical severity was associated with increasing histological scores were assessed for significance by ordinal logistic regression and estimation of odds ratios using the clm() function within the ‘ordinal’ package of R.

To assess whether histological score differed between scorers for the same animals, data were analysed by a mixed effects model within R using the lmer() function within the ‘LmerTest’ package. Scorer was defined as a fixed effect whilst animal was defined as a random factor to account for the repeated measure structure in the data. No account was taken of clinical severity category. Contrasts between scorers were calculated and assessed for significance using the pairs () function of the ‘emmeans’ package of R.

Additionally, the following measures of between rater agreement were calculated, within R to assess overall agreement in scores between users; Average joint probability of agreement & Average Pearson correlation coefficient using the stats() package, Fleiss Kappa, using the irr() package and the Intraclass correlation coefficient using the psych() package.

## Results

### The scoring system uses defined categories to precisely determine GvHD severity

The scoring system includes seven clinical signs; each divided into severity levels. Each level is described and assigned a score, with higher scores indicating greater severity (**Table 1**). The scores are totalled to determine the overall score for the animal. This overall score indicates the overall severity for the animal which is divided into 4 categories: minimal (overall scores of 0-3), mild (overall scores of 4-6), moderate (overall scores of 7-8) and severe (overall scores of ≥ 9) with clear welfare actions assigned to each category, including immediate humane endpoint upon reaching a severe score (**Table 2**). For example, in **Figure 1A** the mouse would be assigned an overall score of 12 (based on scores of 3 for activity, 4 for posture, 1 for inflammation and 4 for fur condition) meaning the mouse would be considered to have reached a severe level and would be culled immediately. In **Figure 1B**, the mouse would be assigned an overall score of 4 (based on scores of 2 for weight loss, 1 for inflammation/reddening of ears, paws, muzzle and tail and 1 for slight piloerection of fur) meaning the mouse would be considered to have reached a mild level and would be scored daily and culled after 14 days (refer to **Tables 1**, **2**). The scoring system has been refined based on our experience with humanised models of cancer to also include clinical signs related to graft-versus-tumour (GvT) responses including yellowing of the tumour and surrounding skin due to proteinaceous oedema resulting from a peri-tumour immune reaction localised to the skin around the tumour (**Figure 1C**). This often coincides with the development of other GvHD clinical signs such as weight loss.

**Figure 2:**
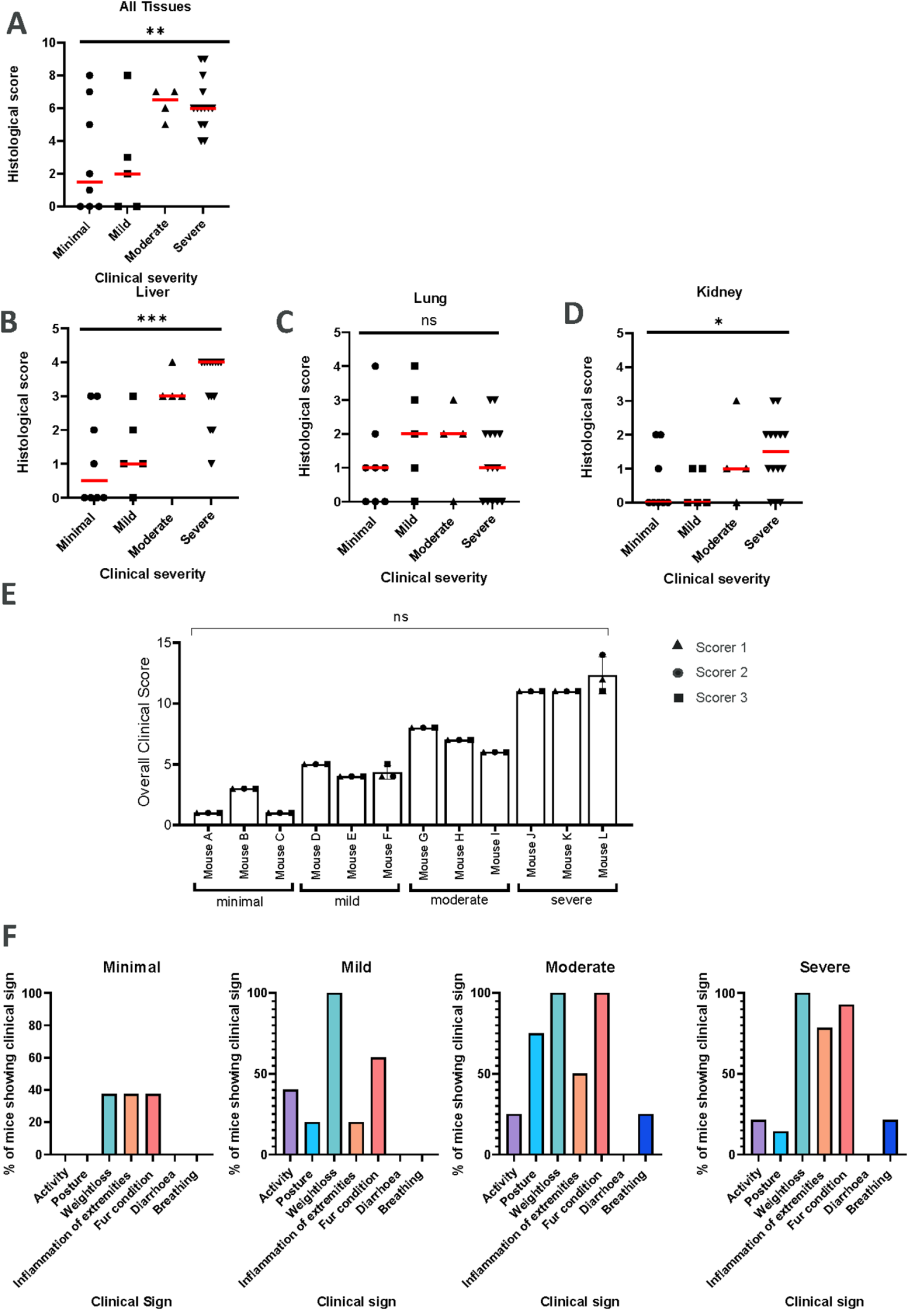
A significant correlation is observed between clinical and histological scores of GvHD. The clinical scoring system is reproducible among different experimenters. **(A)**. Graph of clinical severity plotted against overall histological score (summation of individual organ histological scores). A significant association was observed between clinical severity category and histological score. Symbols represent individual mice, bar indicates median. **P <0.01, ordinal logistic regression and estimation of odds ratio (1.536). **(B–D)**. Graphs of clinical severity plotted against histological scores for each individual organ (liver, lung and kidney). Ordinal logistic regression identified a significant association between clinical severity category and histological score for liver and kidney, but not for the lung. Symbols represent individual mice, bar indicates median. ***P<0.001, *P<0.05, ns, not significant. **(E)**. Three experimenters scored n=3 mice per severity category using the GvHD scoring system. Symbols represent an independent experimenter’s score of each mouse. Mixed effects modelling demonstrates no significant (ns) difference between users across mice and severity levels. **(F)**. Percentage of mice showing each clinical sign of GvHD in the four severity categories from the mice used in this study. Bar graphs are representative of n=8 minimal, n=5 mild, n=4 moderate and n=14 severe mice.

The granularity of our scoring system enabled us to capture the clinical progression of GvHD (**Supplementary Figure 1**), with an increase in the variety of clinical signs observed as the overall severity of GvHD increased (**Figure 2F**). Identifying the predominant clinical signs of GvHD within each severity level is important to better predict welfare outcomes, as well as to improve consistency and uniformity across scorers. We have determined that in cases of minimal GvHD, the most frequently observed clinical signs were minimal weight loss, inflammation and changes in fur condition, all occurring with equal prevalence as percentages of signs seen of all mice in this category (37.5%). In cases of mild GvHD, mild weight loss was the predominant clinical sign (100%), followed by changes in fur condition (60%) and reduced activity (40%). Moderate and severe GvHD presented a broader range of clinical signs, with marked weight loss in all mice changes in fur condition (92%) and inflammation of extremities (78%) being the most common, and breathing abnormalities also becoming evident (25%). Severe GvHD showed all mice having changes in fur conditions and marked weight loss, a higher percentage with inflammation of extremities (78%) and some breathing abnormalities (21%) (**Figure 2F**). The progressive nature of weight loss in these models is managed by adding diet supplements to mice with weight loss greater than 8% from maximum weight in the study (**Supplementary Figure 2**).

### The scoring system is reproducible among different users

To evaluate the consistency of the scoring system, three scientists independently scored the same mice, with three mice coming from each GvHD severity category. The scoring system demonstrated high reproducibility between scientists, with very similar clinical scores observed in each severity category (**Figure 2E**). Additionally, across a range of -r metrics, user scores across mice and severity levels were found to be in high agreement.

### The scoring system accurately captures GvHD clinical signs as it broadly reflects the severity of GvHD-mediated histopathological changes

To establish whether a correlation existed between the overall clinical severity determined by our scoring system and histopathological features of GvHD, mice displaying different severity categories of GvHD were culled and key organs known to be affected by GvHD (liver, kidney and lung) were analysed by a pathologist who was blinded to the clinical score. Across the severity categories, the presence and severity of GvHD-mediated histological changes within the different tissues of individual mice was variable. However, **Figure 2A** revealed a significant association between the overall severity and the total histological scores for all tissues (liver, kidney and lung summed) with GvHD histological scores increasing with increasing clinical severity (p<0.01).

On an individual tissue level, a clear association between clinical and histological scores was observed for liver (p < 0.001), with higher histological scores correlating with greater severity (**Figure 2B**). Similarly, in the kidney, clinical score ranked with histological (p<0.05; **Figure 2D**). Conversely, in the lung, no significant association was observed (**Figure 2C**). These data indicate that the clinical scoring system can broadly reflect the underlying histopathology associated with GvHD.

Tissue changes attributed to GvHD included the following: In the kidney, the changes consisted of an infiltrate of mixed immune cells with a predominance of lymphocytes, present mostly in the renal pelvis area and around arcuate arterioles at the cortico-medullary junction. In the liver, the changes consisted of mixed immune cell infiltration with lymphocytic predominance, present mostly in the portal areas and extending through the limiting plate into the hepatic lobular parenchyma, but also surrounding centrilobular veins, and additionally as a diffuse increase of immune cellularity in hepatic sinusoids. Periportal and peri-centrilobular immune infiltrates were associated with presence of occasional single necrotic hepatocytes. In the lung, GvHD-related changes consisted of a perivascular and sometimes peribronchiolar immune cell infiltration, predominantly lymphocytic, often with extension into the interstitium of surrounding alveolar parenchyma, and rarely within alveolar lumina. The blood vessels surrounded by immune infiltration occasionally showed endothelial hypertrophy, subintimal oedema, and subintimal and mural immune cell infiltrates (**Figure 1E**).

## Discussion

This GvHD scoring system offers an important improvement in the assessment and welfare management of GvHD in humanised mouse models, addressing key limitations of existing systems. Current scoring approaches often fail to capture the multifaceted nature of GvHD-induced clinical signs, and this is particularly important in the early and mild stages when effective intervention is critical to allow mice to remain on studies where the aim is not GvHD assessment but the use of humanised mouse models to assess cancer immunotherapies. In contrast, the system offers a precise, comprehensive, and standardised tool for evaluating clinical signs of GvHD, enabling better animal welfare management and improved study outcomes.

As the use of humanised mouse models in oncology research is rising, the scoring system is part of a broader refinement strategy to address GvHD and welfare concerns in these models. It aids in identifying donors that trigger GvHD soon after humanisation to avoid their use in future studies. Additionally, the NSG MHC I/II DKO strain is used to delay the onset and manifestation of GvHD following humanisation with PBMCs (10). The humanisation method is also considered as mice humanised with *in vitro* expanded T cells typically display less GvHD clinical signs than those humanised with PBMCs. These approaches reduce the need for repeat experiments and minimise overall animal use, aligning with the refinement and reduction principles of the 3Rs and regulatory guidelines, to ensure ethical and responsible research practices.

A key strength of the scoring system lies in its ability to generate clinical scores that correlate with histopathological changes seen in relevant organs such as the liver and kidney, which are consistent with published findings on GvHD histology (3, 5, 6). This alignment between clinical scores and tissue pathology is crucial, as histopathological analysis is considered the gold standard for assessing GvHD severity. The strong correlation demonstrates the scoring system’s validity in capturing the underlying pathology of GvHD. Consequently, this suggests that our scoring system can serve as a reliable alternative to histopathological analysis for tracking GvHD progression, offering a less invasive and more practical approach to monitoring the disease. Interestingly, no significant association was observed in lung pathology, which may reflect organ-specific differences in GvHD progression or immune infiltration or may relate to different humanisation methods or donors. It would be intriguing to investigate if humanised mice exhibiting laboured breathing have increased T cell infiltration and/or more extensive tissue pathology. Indeed, published reports vary on the extent of lung damage observed across different humanised models (17). These findings highlight the complexity of GvHD in humanised models and the need for tailored scoring frameworks that account for model variability.

The presented scoring system demonstrates robust differentiation between minimal, mild, moderate and severe GvHD clinical manifestations as the categories align closely with GvHD histology and therefore underlying biology of GvHD. The system’s design, incorporating a wide range of clinical signs, is particularly suited to the early manifestations and the progressive nature of GvHD observed in tumour-bearing humanised mice. This enables more precise tracking of disease progression particularly in the early stages of GvHD, whereby having two categories (minimal and mild) helps better track early progression enabling early intervention. Whilst some clinical signs, such as weight loss, may be misattributed to GvHD instead of other factors like tumour burden, humanisation or treatment effects, the comprehensive nature of this scoring system predominantly allows for accurate distinction between GvHD and other welfare issues. Our analysis of a broad dataset supports the conclusion that this scoring system is generalisable across various humanised mouse models, irrespective of drug effects, tumour type, strain, sex or humanisation method. However, future work will allow this to be explored further. This versatility makes it highly applicable in large-scale drug discovery settings. Moreover, the system’s successful application in immunotherapy studies underscores its value as a tool for assessing potential GvHD exacerbation by these therapies (18).

The system’s reproducibility across independent experimenters further demonstrates its robustness and utility in large-scale drug discovery settings. By identifying common clinical signs at each severity level, such as weight loss, fur condition changes, and postural abnormalities, the system ensures consistency in scoring among different users. This facilitates training, improves comparability between experiments and reduces variability in data collection. Additionally, the scoring system enables detailed record-keeping and monitoring of GvHD prevalence, allowing for accurate estimation of attrition rates to support power calculations for optimal study design and data collection. The distinct severity categories also permit the calculation of attrition values based on the stage of GvHD at the endpoint of previous studies. Consequently, future studies can be more accurately timed and confidently extended based on this evidence. Furthermore, in large animal units where multiple models are used, the implementation of clear termination criteria is essential to maintain welfare standards.

The scoring system has been developed over the course of several years to incorporate a range of clinical sign categories and terminology to improve ease of use. For example, yellowing of the tumour and surrounding skin, indicative of a GvT reaction, was incorporated based on histological findings that there is a proteinaceous oedema resulting from a peri-tumour immune reaction and/or a GvHD type reaction localised to the skin around the tumour. Severity classifications have been improved by adding clinical signs such as paleness of the extremities and mild breathing abnormalities. Diarrhoea is included in the scoring system despite not being observed in our models because consultations with welfare experts and a review of the literature (19) indicated it would ensure the scoring system remains applicable to a wider variety of humanised mouse models where diarrhoea may occur. By adapting the scoring system over time, we have attempted to encompass all current knowledge of humanised mouse models, thereby refining scoring and improving our animal welfare decisions.

The proposed GvHD scoring system provides a robust, reproducible, and ethically aligned framework for assessing GvHD in humanised mouse models. By improving animal welfare, standardising decision-making, and supporting study continuity, it offers a transformative tool for using humanised mouse studies in large-scale pharmaceutical drug discovery. Continued efforts to refine the scoring system will further enhance its value and applicability across diverse research settings.

## Data Availability

The original contributions presented in the study are included in the article/**Supplementary Material**. Further inquiries can be directed to the corresponding author/s.
