# Supplementary material for: A comprehensive welfare scoring system for graft versus host disease clinical assessment in humanised mouse models used for pharmaceutical research
Source: Front Immunol. 2025 Jun 18;16:1617528. doi: 10.3389/fimmu.2025.1617528 (PMC12213481; doi:10.3389/fimmu.2025.1617528)
Supplement: Supplementary file 1 [file DataSheet1.docx]

**Supplementary Material**

**A comprehensive welfare scoring system for Graft Versus Host Disease (GvHD) clinical assessment in humanised mouse models used for pharmaceutical research**

Alice Nowak, Rebecca Marlow, Kelli Ryan, Jean-Martin Lapointe, Daniel Sutton, Alan Sharpe, Lucy Crook, Jennifer A. Walker, Emma Little, John Peverill, Adam Holberry-Brown, Emma Wassell, Robbie McLaren-Jones, Chelsea Cavanagh, Alex Vlad Dobre, Tamara Baker, Matthew Clayton, Natasha A. Karp, Michiel Plugge, Aurélie A. Thomas, Simon J. Dovedi, Suzanne I. Sitnikova, Natalie Burrows

**Supplementary methods**

**Humanised mouse models of human cancer**

To generate in vitro expanded T cells, human T cells were isolated from human PBMCs and activated with CD3/CD28/CD2 T cell activator kit (Stemcell, 10990) and 50 IU/mL interleukin 2 (IL-2; Roche, 11147528001) for 3 days in ImmunoCult XF T cell expansion Medium (Stemcell, 10981). T cells were then expanded and maintained in media supplemented with 50 IU/mL of IL-2 for 7-9 days (medium was refreshed every 2 days). For PBMC-humanised mice, frozen PBMCs were defrosted and counted. To generate antigen-specific CD8+ T cells, antigen-specific CD8+ T cells were enriched within PBMCs by expanding them for 10 days with an antigen-specific peptide and 50 IU/mL IL-2.

For tumour implantation, mice were shaved on the right flank and subcutaneously injected with 100µL tumour cells (under inhaled isoflurane anaesthesia) containing between 1×10^6^ to 1x10^7^ cells in 1:1 ratio of basement membrane extract (Cultrex (R&D Systems) or Matrigel (Corning)) and phosphate-buffered saline (PBS). Tumour volume was measured three times per week using electronic callipers and calculated using the formula volume=(pi/6)*l*w^2^. Mice were culled due to GvHD (based on severity scores and duration) or upon reaching other predefined endpoints, including humane welfare limits related to tumour volume (≥10% of body weight) or condition (skin ulceration above tumour).

**Supplementary figures**

**Supplementary Figure 1.** Graph of daily recorded clinical severity plotted against days post humanisation of mice that developed severe GvHD (n=13).

**Supplementary Figure 2.** Graph of percentage weight loss of mice as a percentage of the maximum weight loss on study plotted against days post tumour cell implantation (n=6). Each line represents an individual mouse. Arrows indicate when the point where diet supplements were added.
